# Di-μ-adipato-κ^4^*O*^1^,*O*^1′^:*O*^6^,*O*^6′^-bis­[(2,2′-di­pyridyl­amine-κ^2^*N*,*N*′)zinc(II)] trihydrate

**DOI:** 10.1107/S2414314624009064

**Published:** 2024-09-20

**Authors:** Fatima Setifi, Zouaoui Setifi, Thang Pham Chien, Mohammad Hadi Al-Douh, Abderezak Addala

**Affiliations:** a Laboratoire de Chimie Ingénierie Moléculaire et Nanostructures (LCIMN) Université Ferhat Abbas Sétif 1 Sétif 19000 Algeria; b Départment de Technologie Faculté de Technologie Université 20 Août 1955-Skikda BP 26 Route d'El-Hadaiek Skikda 21000 Algeria; c Department of Inorganic Chemistry, Faculty of Chemistry, VNU University of Science, Vietnam National University, Hanoi, 19 Le Thanh Tong, Hanoi, Vietnam; d Chemistry Department, Faculty of Science, Hadhramout University, Mukalla, Hadhramout, Yemen; Vienna University of Technology, Austria

**Keywords:** crystal structure, zinc(II) complexes, 2,2′-di­pyridyl­amine, adipate dianion, octa­hedral coordination

## Abstract

The title compound, [Zn_2_(C_6_H_8_O_4_)_2_(C_10_H_9_N_3_)_2_]·3H_2_O or {Zn_2_[(C_5_H_4_N)_2_NH]_2_[μ-(CH_2_)_4_(COO)_2_]_2_}·3H_2_O, was separ­ated from the solvothermal reaction of zinc(II) sulfate hepta­hydrate, 2,2′-di­pyridyl­amine and sodium adipate. The dinuclear metal complex has a centrosymmetric structure, with the Zn^II^ atom adopting a highly distorted octa­hedral coordination sphere composed of four oxygen atoms from bridging adipato ligands and two pyridine nitro­gen atoms. In the crystal, the title compound aggregates into a tri-periodic supra­molecular structure through inter­molecular hydrogen-bonding networks of the form O—H⋯O and N—H⋯O.

## Abstract 2

The title dinuclear and centrosymmetric complex mol­ecule exhibits a highly distorted octa­hedral O_4_N_2_ coordination set about the central zinc(II) atom.

## Graphical abstract

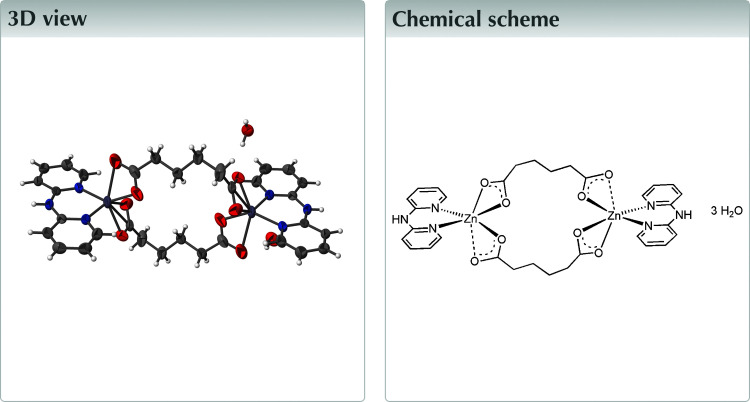

## Structure description

Polynitrile and bis-carboxyl­ate compounds derived from transition-metal ions are of great inter­est with respect to their magnetic and luminescence properties, diverse mol­ecular structures and for their topologies (Addala *et al.* 2019 ▸; Benmansour *et al.*, 2010 ▸; Klongdee *et al.*, 2023 ▸).

As a part of our continuing studies of the structural, magnetic and luminescence properties of coordination complexes containing polynitrile and/or bis-carboxyl­ate and polypyridyl units (Setifi *et al.*, 2006 ▸, 2016 ▸; Lehchili *et al.*, 2017 ▸), we report here the mol­ecular and crystal structure of the dinuclear compound, [Zn_2_(adp)_2_(dpa)_2_]·3H_2_O, based on the adipate dianion (adp) as ligand and the 2,2′-di­pyridyl­amine (dpa) as co-ligand.

**Figure 1:**
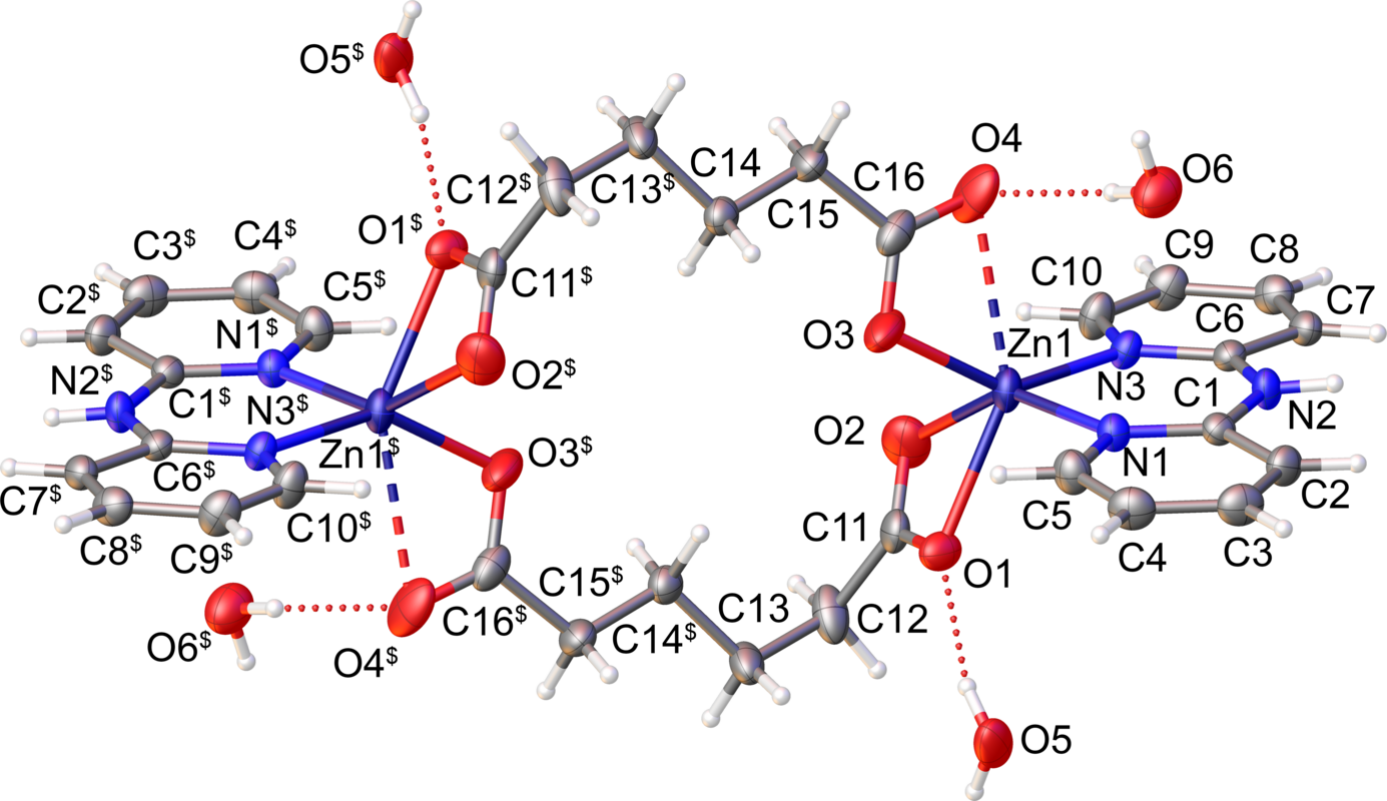
The mol­ecular structure of the title compound showing the atom labeling. Displacement ellipsoids are drawn at the 50% probability level. [Symmetry code: ($) −*x* + 1, −*y* + 1, −*z* + 1.]

The asymmetric unit of the title compound consists of half of the metal complex mol­ecule and two water mol­ecules (one of which exhibits half-occupancy). The mol­ecule is completed by inversion symmetry. The Zn^II^ atom is octa­hedrally coordinated by two (*O*,*O′*) donor sets of carboxyl­ato groups from two different adp ligands and one (*N*,*N*)-chelating dpa co-ligand (Fig. 1 ▸). Except the Zn—O4 contact with a significantly long bond of 2.567 (2) Å, the lengths of other Zn—O bonds are in the range of 2.0073 (19)–2.2146 (17) Å, while Zn1—N1 and Zn1—N3 bond lengths are 2.0473 (16) and 2.0470 (16) Å, respectively. As a result of the long Zn—O4 bond, the highly distorted octa­hedral coordination environment of Zn^II^ ion is best described as [5 + 1]. This bonding situation of the present dinuclear Zn^II^ compound closely resembles Ni^II^ and Cu^II^ counterparts previously reported (Setifi *et al.*, 2014 ▸).

**Figure 2:**
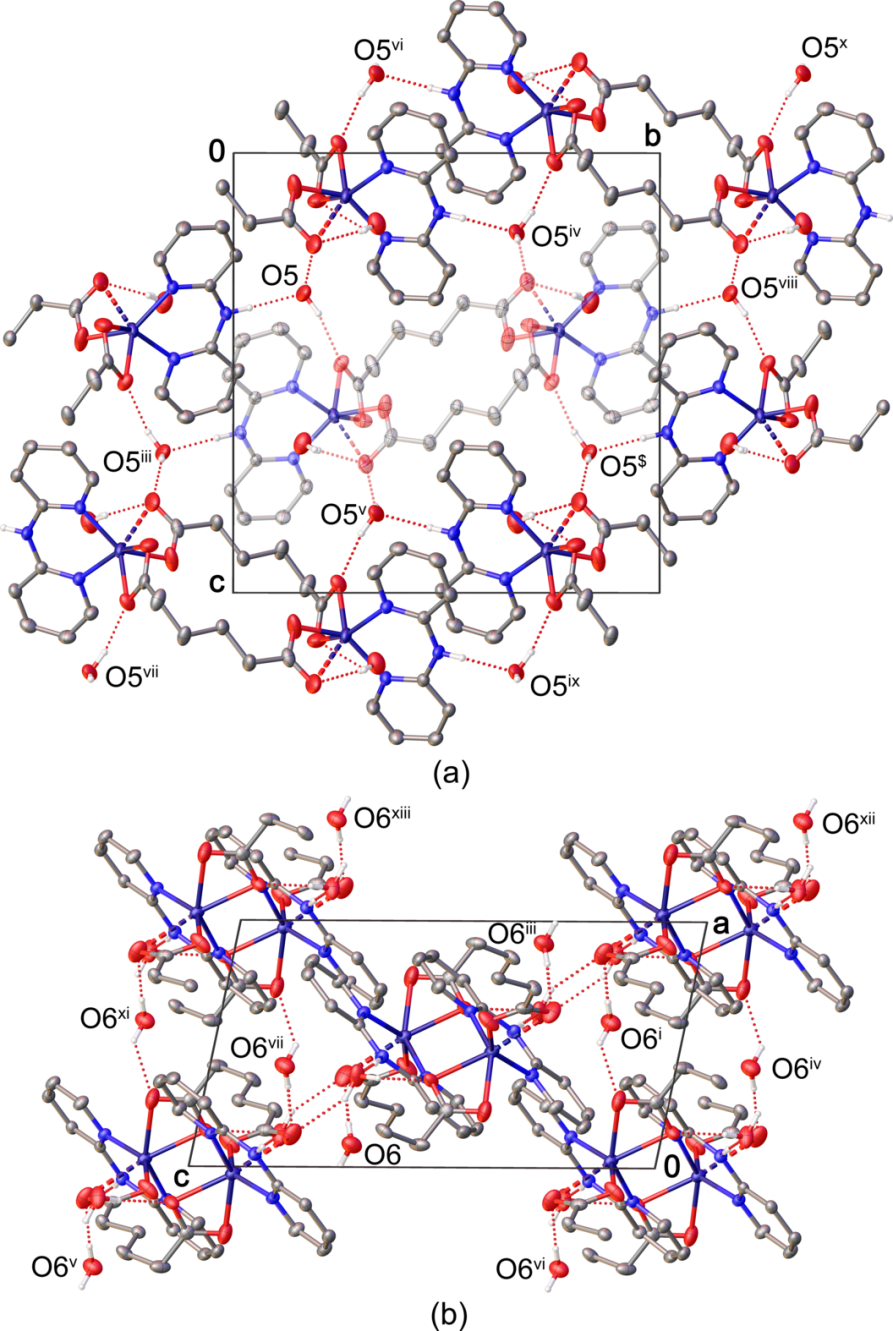
Partial packing diagram showing the hydrogen-bonding inter­actions involving (*a*) O5 water mol­ecules (shown in projection down the *a* axis) and (*b*) O6 water mol­ecules (shown in projection down the *b* axis). Hydrogen atoms bonded to carbon atoms were omitted for clarity. [Symmetry codes: (i) *x* + 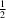, −*y* + 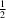, *z* − 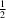; (iii) −*x* + 1, −*y*, −*z* + 1; (iv) −*x* + 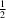, *y* + 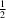, −*z* + 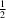; (v) *x* − 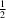, −*y* + 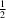, *z* + 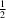; (vi) *x* − 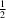, −*y* + 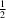, *z* − 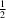; (vii) −*x* + 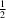, *y* − 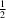, −*z* + 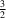; (viii) *x*, *y* + 1, *z*; (ix) −*x* + 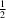, *y* + 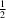, −*z* + 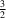; (*x*) *x* + 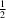, −*y* + 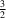, *z* − 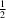; (xi) *x* + 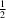, −*y* + 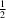, *z* + 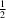; (xii) −*x* + 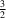, *y* + 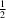, −*z* + 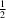; (xiii) −*x* + 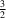, *y* + 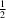, −*z* + 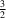].

In the crystal, complex mol­ecules aggregate into a tri-periodic supra­molecular hydrogen-bonding network. The NH groups of dpa form hydrogen bonds with the O5 water mol­ecules, which in turn provide hydrogen bonds to carboxyl­ate oxygen atoms (O1 and O4) of adp (Table 1 ▸, Fig. 2 ▸*a*). In addition, the half-occupied O6 water mol­ecule is also involved in weaker hydrogen bonds with adp through O2 and O4 atoms (Table 1 ▸, Fig. 2 ▸*b*).

## Synthesis and crystallization

The title compound was prepared solvothermally under autogenous pressure from a mixture of zinc(II) sulfate hepta­hydrate (29 mg, 0.1 mmol), 2,2′-di­pyridyl­amine (24 mg, 0.2 mmol) and sodium adipate (0.2 mol l^−1^) in a mixture of water and ethanol (4:1 *v*/*v*, 25 ml). This mixture was sealed in a Teflon-lined autoclave and held at 393 K for 2 d, and then cooled to ambient temperature at a rate of 10 K h^−1^ to give the product in form of colorless needles (yield 36%).

## Refinement

Crystal data, data collection and structure refinement details are summarized in Table 2 ▸. The O6 atom and associated H atoms of the second water mol­ecule were refined with an occupancy of 0.5. Hydrogen atoms of the water mol­ecules were included in calculated positions and were refined in a riding model, with O—H distances of 0.85 Å and *U*_iso_(H) = 1.5*U*_eq_(O).

## Supplementary Material

Crystal structure: contains datablock(s) I. DOI: 10.1107/S2414314624009064/wm4220sup1.cif

Structure factors: contains datablock(s) I. DOI: 10.1107/S2414314624009064/wm4220Isup2.hkl

CCDC reference: 2384469

Additional supporting information:  crystallographic information; 3D view; checkCIF report

## Figures and Tables

**Table 1:** Hydrogen-bond geometry (Å, °)

*D*—H⋯*A*	*D*—H	H⋯*A*	*D*⋯*A*	*D*—H⋯*A*
O5—H5*A*⋯O1	0.85	1.89	2.728 (3)	167
O5—H5*B*⋯O4^i^	0.85	2.10	2.877 (3)	151
O6—H6*A*⋯O4	0.85	2.26	3.107 (5)	173
O6—H6*B*⋯O2^ii^	0.85	2.58	3.275 (5)	140
N2—H2⋯O5^iii^	0.86	1.97	2.824 (2)	177

Symmetry codes: (i) 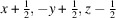; (ii) 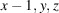; (iii) 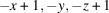.

**Table 2:** Experimental details

Crystal data	
Chemical formula	[Zn_2_(C_6_H_8_O_4_)_2_(C_10_H_9_N_3_)_2_]·3H_2_O
*M*_r_	815.44
Crystal system, space group	Monoclinic, *P*2_1_/*n*
Temperature (K)	302
*a*, *b*, *c* (Å)	8.2105 (3), 14.4478 (6), 15.2042 (8)
β (°)	101.694 (2)
*V* (Å^3^)	1766.14 (14)
*Z*	2
Radiation type	Mo *K*α
μ (mm^−1^)	1.43
Crystal size (mm)	0.18 × 0.11 × 0.06

Data collection	
Diffractometer	Oxford Diffraction Xcalibur with Sapphire CCD
Absorption correction	Multi-scan (*CrysAlis RED*; Oxford Diffraction, 2009 ▸)
*T*_min_, *T*_max_	0.492, 1.000
No. of measured, independent and observed [*I* > 2σ(*I*)] reflections	20158, 6416, 4033
*R*_int_	0.038
(sin θ/λ)_max_ (Å^−1^)	0.762

Refinement	
*R*[*F*^2^ > 2σ(*F*^2^)], *wR*(*F*^2^), *S*	0.044, 0.102, 1.02
No. of reflections	6416
No. of parameters	241
H-atom treatment	H-atom parameters constrained
Δρ_max_, Δρ_min_ (e Å^−3^)	0.48, −0.86

Computer programs: *CrysAlis CCD* (Oxford Diffraction, 2009 ▸), *CrysAlis RED* (Oxford Diffraction, 2009 ▸), *SHELXT2014/5* (Sheldrick, 2015*a* ▸), *SHELXL2018/3* (Sheldrick, 2015*b* ▸), *OLEX2* 1.5 (Dolomanov *et al.*, 2009 ▸) and *publCIF* (Westrip, 2010 ▸).

## full crystallographic data

### Di-µ-adipato-κ4O1,O1':O6,O6'-bis{[N-(pyridin-2-yl-κN)pyridin-2-amine-κN1]zinc(II)} trihydrate Crystal data

**Table:**

[Zn_2_(C_6_H_8_O_4_)_2_(C_10_H_9_N_3_)_2_]·3H_2_O	*F*(000) = 844
*M**_r_* = 815.44	*D*_x_ = 1.533 Mg m^−^^3^
Monoclinic, *P*2_1_/*n*	Mo *K*α radiation, λ = 0.71073 Å
*a* = 8.2105 (3) Å	Cell parameters from 1439 reflections
*b* = 14.4478 (6) Å	θ = 2.3–26.3°
*c* = 15.2042 (8) Å	µ = 1.43 mm^−^^1^
β = 101.694 (2)°	*T* = 302 K
*V* = 1766.14 (14) Å^3^	Needle, colourless
*Z* = 2	0.18 × 0.11 × 0.06 mm

### Di-µ-adipato-κ4O1,O1':O6,O6'-bis{[N-(pyridin-2-yl-κN)pyridin-2-amine-κN1]zinc(II)} trihydrate Data collection

**Table:**

Oxford Diffraction Xcalibur with Sapphire CCD diffractometer	4033 reflections with *I* > 2σ(*I*)
Radiation source: Enhance (Mo) X-ray source	*R*_int_ = 0.038
ω scans	θ_max_ = 32.8°, θ_min_ = 3.6°
Absorption correction: multi-scan (Crysalis Red; Oxford Diffraction, 2009)	*h* = −10→12
*T*_min_ = 0.492, *T*_max_ = 1.000	*k* = −22→21
20158 measured reflections	*l* = −23→21
6416 independent reflections	

### Di-µ-adipato-κ4O1,O1':O6,O6'-bis{[N-(pyridin-2-yl-κN)pyridin-2-amine-κN1]zinc(II)} trihydrate Refinement

**Table:**

Refinement on *F*^2^	Primary atom site location: dual
Least-squares matrix: full	Hydrogen site location: mixed
*R*[*F*^2^ > 2σ(*F*^2^)] = 0.044	H-atom parameters constrained
*wR*(*F*^2^) = 0.102	*w* = 1/[σ^2^(*F*_o_^2^) + (0.0328*P*)^2^ + 0.9459*P*] where *P* = (*F*_o_^2^ + 2*F*_c_^2^)/3
*S* = 1.02	(Δ/σ)_max_ = 0.001
6416 reflections	Δρ_max_ = 0.48 e Å^−^^3^
241 parameters	Δρ_min_ = −0.86 e Å^−^^3^
0 restraints	

### Di-µ-adipato-κ4O1,O1':O6,O6'-bis{[N-(pyridin-2-yl-κN)pyridin-2-amine-κN1]zinc(II)} trihydrate Special details

**Table:**

Geometry. All e.s.d.'s (except the e.s.d. in the dihedral angle between two l.s. planes) are estimated using the full covariance matrix. The cell e.s.d.'s are taken into account individually in the estimation of e.s.d.'s in distances, angles and torsion angles; correlations between e.s.d.'s in cell parameters are only used when they are defined by crystal symmetry. An approximate (isotropic) treatment of cell e.s.d.'s is used for estimating e.s.d.'s involving l.s. planes.

### Di-µ-adipato-κ4O1,O1':O6,O6'-bis{[N-(pyridin-2-yl-κN)pyridin-2-amine-κN1]zinc(II)} trihydrate Fractional atomic coordinates and isotropic or equivalent isotropic displacement parameters (Å^2^)

**Table:**

	*x*	*y*	*z*	*U*_iso_*/*U*_eq_	Occ. (<1)
Zn1	0.52923 (3)	0.23563 (2)	0.59713 (2)	0.03924 (9)	
O4	0.3690 (3)	0.31102 (13)	0.70690 (14)	0.0791 (7)	
O2	0.7724 (3)	0.30383 (15)	0.60833 (13)	0.0752 (6)	
O1	0.6465 (2)	0.25296 (11)	0.47921 (12)	0.0498 (4)	
O5	0.6289 (3)	0.16459 (13)	0.31903 (14)	0.0725 (6)	
H5A	0.639294	0.199470	0.364850	0.109*	
H5B	0.722914	0.167391	0.303780	0.109*	
O6	0.0902 (5)	0.1631 (3)	0.6599 (3)	0.0730 (11)	0.5
H6A	0.171671	0.199984	0.675684	0.109*	0.5
H6B	0.008471	0.190701	0.674841	0.109*	0.5
N1	0.3655 (2)	0.13927 (11)	0.53175 (11)	0.0347 (4)	
N2	0.4477 (2)	0.01757 (11)	0.63553 (12)	0.0375 (4)	
H2	0.420818	−0.037731	0.647937	0.045*	
N3	0.6264 (2)	0.14173 (11)	0.69451 (11)	0.0352 (4)	
O3	0.4049 (3)	0.35591 (12)	0.57654 (16)	0.0757 (6)	
C1	0.3528 (2)	0.05144 (13)	0.55665 (13)	0.0320 (4)	
C2	0.2422 (3)	−0.01017 (15)	0.50387 (15)	0.0423 (5)	
H2A	0.235752	−0.071351	0.521863	0.051*	
C3	0.1439 (3)	0.02034 (18)	0.42585 (16)	0.0488 (6)	
H3	0.069354	−0.019799	0.390602	0.059*	
C4	0.1559 (3)	0.11133 (18)	0.39960 (16)	0.0503 (6)	
H4	0.090285	0.133564	0.346685	0.060*	
C5	0.2670 (3)	0.16751 (16)	0.45378 (16)	0.0462 (5)	
H5	0.275555	0.228659	0.436171	0.055*	
C6	0.5767 (2)	0.05380 (13)	0.69865 (13)	0.0315 (4)	
C7	0.6539 (3)	−0.00637 (14)	0.76730 (14)	0.0369 (4)	
H7	0.618034	−0.067305	0.768749	0.044*	
C8	0.7820 (3)	0.02557 (16)	0.83172 (15)	0.0434 (5)	
H8	0.834264	−0.013415	0.877486	0.052*	
C9	0.8335 (3)	0.11660 (16)	0.82839 (16)	0.0485 (5)	
H9	0.919951	0.139854	0.871929	0.058*	
C10	0.7542 (3)	0.17143 (15)	0.75964 (16)	0.0456 (5)	
H10	0.789670	0.232347	0.757327	0.055*	
C16	0.3524 (3)	0.36974 (15)	0.64708 (19)	0.0480 (6)	
C15	0.2705 (3)	0.46125 (15)	0.65891 (17)	0.0473 (5)	
H15A	0.341863	0.494613	0.707215	0.057*	
H15B	0.167041	0.448608	0.678170	0.057*	
C14	0.2323 (3)	0.52446 (14)	0.57801 (15)	0.0403 (5)	
H14A	0.161271	0.492051	0.528875	0.048*	
H14B	0.335121	0.539790	0.559240	0.048*	
C13	0.8531 (3)	0.38678 (15)	0.40257 (19)	0.0519 (6)	
H13A	0.949705	0.402554	0.377984	0.062*	
H13B	0.777494	0.351921	0.357227	0.062*	
C12	0.9082 (3)	0.32560 (18)	0.4846 (2)	0.0611 (7)	
H12A	0.965667	0.272054	0.467267	0.073*	
H12B	0.986835	0.359756	0.529059	0.073*	
C11	0.7690 (3)	0.29305 (14)	0.52654 (16)	0.0420 (5)	

### Di-µ-adipato-κ4O1,O1':O6,O6'-bis{[N-(pyridin-2-yl-κN)pyridin-2-amine-κN1]zinc(II)} trihydrate Atomic displacement parameters (Å^2^)

**Table:**

	*U*^11^	*U*^22^	*U*^33^	*U*^12^	*U*^13^	*U*^23^
Zn1	0.04188 (14)	0.02539 (11)	0.04678 (16)	−0.00240 (9)	0.00029 (10)	0.00485 (10)
O4	0.1090 (17)	0.0462 (11)	0.0712 (14)	0.0344 (11)	−0.0072 (12)	0.0039 (9)
O2	0.1084 (17)	0.0698 (13)	0.0417 (11)	−0.0076 (12)	0.0020 (10)	0.0048 (9)
O1	0.0475 (8)	0.0476 (9)	0.0585 (11)	−0.0147 (7)	0.0204 (8)	−0.0152 (7)
O5	0.1197 (18)	0.0454 (10)	0.0540 (12)	−0.0324 (11)	0.0216 (11)	−0.0117 (8)
O6	0.068 (3)	0.060 (2)	0.095 (3)	−0.0104 (19)	0.027 (2)	−0.015 (2)
N1	0.0350 (8)	0.0316 (8)	0.0364 (9)	−0.0025 (6)	0.0044 (7)	0.0024 (7)
N2	0.0445 (9)	0.0270 (8)	0.0396 (10)	−0.0074 (7)	0.0050 (7)	0.0037 (7)
N3	0.0383 (8)	0.0266 (7)	0.0388 (9)	0.0020 (6)	0.0028 (7)	−0.0011 (6)
O3	0.0923 (14)	0.0387 (9)	0.1157 (18)	−0.0024 (9)	0.0674 (14)	−0.0157 (10)
C1	0.0309 (9)	0.0323 (9)	0.0342 (10)	−0.0020 (7)	0.0100 (7)	−0.0012 (7)
C2	0.0439 (11)	0.0360 (10)	0.0468 (13)	−0.0097 (8)	0.0090 (9)	−0.0051 (9)
C3	0.0393 (11)	0.0579 (14)	0.0463 (14)	−0.0089 (10)	0.0022 (9)	−0.0121 (11)
C4	0.0464 (12)	0.0623 (15)	0.0379 (13)	0.0038 (11)	−0.0015 (10)	0.0010 (11)
C5	0.0489 (12)	0.0428 (12)	0.0432 (13)	−0.0014 (9)	0.0011 (10)	0.0067 (9)
C6	0.0338 (9)	0.0289 (9)	0.0336 (10)	0.0031 (7)	0.0112 (7)	−0.0011 (7)
C7	0.0436 (11)	0.0317 (9)	0.0374 (11)	0.0049 (8)	0.0128 (8)	0.0034 (8)
C8	0.0459 (12)	0.0439 (12)	0.0389 (12)	0.0116 (9)	0.0052 (9)	0.0050 (9)
C9	0.0479 (12)	0.0467 (13)	0.0443 (13)	0.0032 (10)	−0.0063 (10)	−0.0024 (10)
C10	0.0490 (12)	0.0327 (10)	0.0486 (13)	−0.0028 (9)	−0.0059 (10)	−0.0032 (9)
C16	0.0352 (10)	0.0348 (11)	0.0721 (17)	−0.0011 (8)	0.0067 (10)	−0.0129 (11)
C15	0.0572 (13)	0.0351 (11)	0.0547 (14)	0.0089 (9)	0.0237 (11)	0.0008 (10)
C14	0.0405 (10)	0.0337 (10)	0.0482 (13)	−0.0049 (8)	0.0126 (9)	−0.0022 (9)
C13	0.0521 (13)	0.0379 (11)	0.0749 (18)	0.0038 (10)	0.0342 (12)	0.0127 (11)
C12	0.0382 (12)	0.0476 (13)	0.097 (2)	−0.0029 (10)	0.0115 (12)	0.0249 (13)
C11	0.0505 (12)	0.0241 (8)	0.0501 (14)	0.0025 (8)	0.0071 (10)	0.0064 (8)

### Di-µ-adipato-κ4O1,O1':O6,O6'-bis{[N-(pyridin-2-yl-κN)pyridin-2-amine-κN1]zinc(II)} trihydrate Geometric parameters (Å, º)

**Table:**

Zn1—O4	2.567 (2)	C3—C4	1.383 (4)
Zn1—O2	2.202 (2)	C4—H4	0.9300
Zn1—O1	2.2146 (17)	C4—C5	1.366 (3)
Zn1—N1	2.0473 (16)	C5—H5	0.9300
Zn1—N3	2.0470 (16)	C6—C7	1.407 (3)
Zn1—O3	2.0073 (19)	C7—H7	0.9300
Zn1—C11	2.563 (2)	C7—C8	1.364 (3)
O4—C16	1.231 (3)	C8—H8	0.9300
O2—C11	1.248 (3)	C8—C9	1.385 (3)
O1—C11	1.253 (3)	C9—H9	0.9300
O5—H5A	0.8500	C9—C10	1.367 (3)
O5—H5B	0.8504	C10—H10	0.9300
O6—H6A	0.8508	C16—C15	1.511 (3)
O6—H6B	0.8500	C15—H15A	0.9700
N1—C1	1.334 (2)	C15—H15B	0.9700
N1—C5	1.355 (3)	C15—C14	1.513 (3)
N2—H2	0.8600	C14—H14A	0.9700
N2—C1	1.381 (2)	C14—H14B	0.9700
N2—C6	1.380 (2)	C14—C13^i^	1.519 (3)
N3—C6	1.340 (2)	C13—H13A	0.9700
N3—C10	1.358 (3)	C13—H13B	0.9700
O3—C16	1.250 (3)	C13—C12	1.520 (3)
C1—C2	1.402 (3)	C12—H12A	0.9700
C2—H2A	0.9300	C12—H12B	0.9700
C2—C3	1.365 (3)	C12—C11	1.493 (3)
C3—H3	0.9300		
			
O2—Zn1—O4	109.63 (8)	C4—C5—H5	118.1
O2—Zn1—O1	58.38 (7)	N2—C6—C7	116.65 (17)
O2—Zn1—C11	29.12 (7)	N3—C6—N2	121.67 (17)
O1—Zn1—O4	147.40 (6)	N3—C6—C7	121.68 (18)
O1—Zn1—C11	29.26 (6)	C6—C7—H7	120.3
N1—Zn1—O4	103.40 (7)	C8—C7—C6	119.32 (19)
N1—Zn1—O2	146.89 (8)	C8—C7—H7	120.3
N1—Zn1—O1	91.96 (6)	C7—C8—H8	120.3
N1—Zn1—C11	120.07 (7)	C7—C8—C9	119.4 (2)
N3—Zn1—O4	89.24 (6)	C9—C8—H8	120.3
N3—Zn1—O2	91.51 (7)	C8—C9—H9	120.8
N3—Zn1—O1	119.40 (7)	C10—C9—C8	118.5 (2)
N3—Zn1—N1	91.24 (6)	C10—C9—H9	120.8
N3—Zn1—C11	107.32 (7)	N3—C10—C9	123.5 (2)
O3—Zn1—O4	54.76 (7)	N3—C10—H10	118.2
O3—Zn1—O2	92.81 (8)	C9—C10—H10	118.2
O3—Zn1—O1	93.82 (7)	O4—C16—O3	121.2 (2)
O3—Zn1—N1	104.48 (8)	O4—C16—C15	120.0 (2)
O3—Zn1—N3	142.92 (8)	O3—C16—C15	118.8 (2)
O3—Zn1—C11	93.66 (7)	C16—C15—H15A	108.0
C11—Zn1—O4	132.29 (7)	C16—C15—H15B	108.0
C16—O4—Zn1	78.85 (16)	C16—C15—C14	117.1 (2)
C11—O2—Zn1	91.72 (15)	H15A—C15—H15B	107.3
C11—O1—Zn1	90.99 (15)	C14—C15—H15A	108.0
H5A—O5—H5B	104.5	C14—C15—H15B	108.0
H6A—O6—H6B	104.4	C15—C14—H14A	109.1
C1—N1—Zn1	126.18 (13)	C15—C14—H14B	109.1
C1—N1—C5	117.85 (18)	C15—C14—C13^i^	112.29 (19)
C5—N1—Zn1	115.89 (14)	H14A—C14—H14B	107.9
C1—N2—H2	113.3	C13^i^—C14—H14A	109.1
C6—N2—H2	113.3	C13^i^—C14—H14B	109.1
C6—N2—C1	133.48 (16)	C14^i^—C13—H13A	108.8
C6—N3—Zn1	125.79 (13)	C14^i^—C13—H13B	108.8
C6—N3—C10	117.58 (17)	C14^i^—C13—C12	113.8 (2)
C10—N3—Zn1	116.62 (13)	H13A—C13—H13B	107.7
C16—O3—Zn1	104.91 (17)	C12—C13—H13A	108.8
N1—C1—N2	121.38 (17)	C12—C13—H13B	108.8
N1—C1—C2	121.34 (18)	C13—C12—H12A	108.7
N2—C1—C2	117.28 (18)	C13—C12—H12B	108.7
C1—C2—H2A	120.2	H12A—C12—H12B	107.6
C3—C2—C1	119.5 (2)	C11—C12—C13	114.03 (19)
C3—C2—H2A	120.2	C11—C12—H12A	108.7
C2—C3—H3	120.2	C11—C12—H12B	108.7
C2—C3—C4	119.6 (2)	O2—C11—Zn1	59.17 (14)
C4—C3—H3	120.2	O2—C11—O1	118.9 (2)
C3—C4—H4	121.0	O2—C11—C12	121.4 (2)
C5—C4—C3	118.0 (2)	O1—C11—Zn1	59.75 (12)
C5—C4—H4	121.0	O1—C11—C12	119.6 (2)
N1—C5—C4	123.8 (2)	C12—C11—Zn1	179.3 (2)
N1—C5—H5	118.1		
			
Zn1—O4—C16—O3	4.8 (2)	C1—N1—C5—C4	0.0 (3)
Zn1—O4—C16—C15	−174.0 (2)	C1—N2—C6—N3	5.6 (3)
Zn1—O2—C11—O1	−0.5 (2)	C1—N2—C6—C7	−174.1 (2)
Zn1—O2—C11—C12	−179.62 (18)	C1—C2—C3—C4	0.6 (3)
Zn1—O1—C11—O2	0.5 (2)	C2—C3—C4—C5	−0.1 (4)
Zn1—O1—C11—C12	179.63 (18)	C3—C4—C5—N1	−0.2 (4)
Zn1—N1—C1—N2	3.8 (3)	C5—N1—C1—N2	−179.40 (19)
Zn1—N1—C1—C2	−176.34 (15)	C5—N1—C1—C2	0.4 (3)
Zn1—N1—C5—C4	177.1 (2)	C6—N2—C1—N1	−7.0 (3)
Zn1—N3—C6—N2	−1.2 (3)	C6—N2—C1—C2	173.1 (2)
Zn1—N3—C6—C7	178.48 (14)	C6—N3—C10—C9	−0.1 (3)
Zn1—N3—C10—C9	−179.1 (2)	C6—C7—C8—C9	0.0 (3)
Zn1—O3—C16—O4	−6.3 (3)	C7—C8—C9—C10	−0.5 (4)
Zn1—O3—C16—C15	172.52 (17)	C8—C9—C10—N3	0.5 (4)
O4—C16—C15—C14	−171.6 (2)	C10—N3—C6—N2	179.91 (19)
N1—C1—C2—C3	−0.7 (3)	C10—N3—C6—C7	−0.4 (3)
N2—C1—C2—C3	179.1 (2)	C16—C15—C14—C13^i^	178.8 (2)
N2—C6—C7—C8	−179.88 (19)	C14^i^—C13—C12—C11	61.0 (3)
N3—C6—C7—C8	0.4 (3)	C13—C12—C11—O2	−127.6 (3)
O3—C16—C15—C14	9.6 (3)	C13—C12—C11—O1	53.3 (3)

Symmetry code: (i) −*x*+1, −*y*+1, −*z*+1.

### Di-µ-adipato-κ4O1,O1':O6,O6'-bis{[N-(pyridin-2-yl-κN)pyridin-2-amine-κN1]zinc(II)} trihydrate Hydrogen-bond geometry (Å, º)

**Table:**

*D*—H···*A*	*D*—H	H···*A*	*D*···*A*	*D*—H···*A*
O5—H5*A*···O1	0.85	1.89	2.728 (3)	167
O5—H5*B*···O4^ii^	0.85	2.10	2.877 (3)	151
O6—H6*A*···O4	0.85	2.26	3.107 (5)	173
O6—H6*B*···O2^iii^	0.85	2.58	3.275 (5)	140
N2—H2···O5^iv^	0.86	1.97	2.824 (2)	177

Symmetry codes: (ii) *x*+1/2, −*y*+1/2, *z*−1/2; (iii) *x*−1, *y*, *z*; (iv) −*x*+1, −*y*, −*z*+1.
